# P-4. The Clinical Characteristics and Outcomes of Combination Therapy on Methicillin-susceptible Staphylococcus aureus Bacteremia: A 5-Year Experience

**DOI:** 10.1093/ofid/ofaf695.235

**Published:** 2026-01-11

**Authors:** Shaikha D Al-Shokri, Myeongji Kim, Omar M Abu Saleh, Nischal Ranganath, Zachary Yetmar, Ryan W W Stevens, Supavit Chesdachai

**Affiliations:** Mayo Clinic, Rochester, Minnesota; Mayo Clinic, Rochester, Minnesota; Mayo Clinic, Rochester, Minnesota; Mayo Clinic, Rochester, Minnesota; Cleveland Clinic, Cleveland, OH; Mayo Clinic, Rochester, Minnesota; Mayo Clinic, Rochester, Minnesota

## Abstract

**Background:**

Despite effective therapy for methicillin-susceptible *Staphylococcus aureus* (MSSA) bacteremia with anti-staphylococcal penicillins (ASP; e.g., oxacillin, nafcillin) or cefazolin, persistent bacteremia is associated with poorer clinical outcomes and increased complications. Emerging strategies, such as combination therapy with an ASP or cefazolin and ertapenem, have demonstrated the potential to accelerate microbiologic clearance in persistent MSSA bacteremia. However, despite its increasing utilization in clinical practice, data on the effectiveness of this combination regimen remains limited. We sought to describe the clinical characteristics and outcomes of patients with MSSA bacteremia receiving combination therapy.

**Methods:**

We performed a single-center, retrospective study of adult patients with MSSA bacteremia who received ASP or cefazolin and ertapenem combination therapy from 2019-2023. Time to blood culture clearance, in-hospital mortality, and 90-day relapse were examined.

**Results:**

A total of 57 patients were included: median age of 65 years [IQR 53-73] with 75.4% male. MSSA bacteremia complicated by infective endocarditis and cardiac implantable electronic device infection occurred in 35.1% and 12.3%, respectively. The median length-of-stay was 16 days [IQR 12-28], and 57.9% required ICU admission. Forty (70.2%) patients underwent source control interventions such as valve replacement, device extraction or surgical debridement. The median duration of bacteremia prior to combination therapy initiation was 4 days [IQR 3-6]. Blood culture clearance occurred in a median of 1 day. The median duration of combination therapy was 4 days [IQR 3-6]. In-hospital mortality was 15.8%. None of the patients surviving the hospitalization experienced relapsed bacteremia within 90 days.

**Conclusion:**

This study supports growing evidence that combination therapy may offer benefits in managing persistent MSSA bacteremia, specifically with regards to time to blood culture clearance. Further investigation is warranted to determine optimal timing of combination therapy and if known benefits of combination therapy translate into superior clinical outcomes as compared to monotherapy.

**Disclosures:**

All Authors: No reported disclosures

